# Optimal Assessment of Nutritional Status in Older Subjects with the Chronic Obstructive Pulmonary Disease—A Comparison of Three Screening Tools Used in the GLIM Diagnostic Algorithm

**DOI:** 10.3390/ijerph19031025

**Published:** 2022-01-18

**Authors:** Aleksandra Kaluźniak-Szymanowska, Roma Krzymińska-Siemaszko, Katarzyna Wieczorowska-Tobis, Ewa Deskur-Śmielecka

**Affiliations:** Department of Palliative Medicine, Poznan University of Medical Sciences, 61-245 Poznan, Poland; krzyminskasiemaszko@ump.edu.pl (R.K.-S.); kwt@tobis.pl (K.W.-T.); edeskur@ump.edu.pl (E.D.-Ś.)

**Keywords:** older adults, malnutrition, COPD, screening tools, GLIM

## Abstract

Chronic obstructive pulmonary disease (COPD) is a recognized risk factor for malnutrition. The European Respiratory Society (ERS) statement included nutritional status assessment and dietary intervention as essential components of comprehensive management in subjects with COPD. According to the GLIM algorithm, the first step in diagnosing malnutrition is risk screening with a validated tool. Our study aimed to assess the diagnostic performance of three screening tools (MNA-SF, MUST, and NRS-2002) used in the GLIM algorithm in older patients with COPD. Additionally, we evaluated the agreement between these tools in the diagnostics of malnutrition. We performed a cross-sectional study of 124 patients aged at least 60 years with COPD diagnosed, based on the Global Initiative for Chronic Obstructive Lung Disease (GOLD). We assessed the participants’ nutritional status with the three examined screening questionnaires (MNA-SF, MUST, and NRS-2002). Regardless of their results, we performed full malnutrition diagnostics following the GLIM algorithm in all subjects. The proportion of malnourished participants varied from 18.5% for the MUST questionnaire to 27.4% for the MNA-SF and 57.3% for the NRS-2002 score. Based on the GLIM criteria, malnutrition was diagnosed in 48 subjects (38.7%). All assessed questionnaires had an unsatisfactory sensitivity against the GLIM criteria for malnutrition: it was fair (58.3%) for the MNA-SF tool and poor for the MUST and NRS-2002 questionnaires (47.9% for both questionnaires). Considering the negative health consequences of malnutrition, a full diagnostic including GLIM etiologic and phenotypic criteria should be recommended in all elderly patients with COPD, regardless of the screening results.

## 1. Introduction

Disease-related malnutrition is found in 20–50% of hospitalized subjects. Chronic obstructive pulmonary disease (COPD) is a recognized risk factor for malnutrition [1,2]. The European Respiratory Society (ERS) statement included nutritional status assessment and dietary intervention as essential components of comprehensive management in subjects with COPD [3]. In the same statement, experts from the ERS pointed out that screening questionnaires such as the Mini Nutritional Assessment (MNA) and Malnutrition Universal Screening Tool (MUST) estimate nutritional status without assessing changes in body composition (e.g., muscle mass loss), common in older patients with COPD [3].

In 2018, experts from the Global Leadership Initiative on Malnutrition (GLIM) launched new diagnostic criteria for malnutrition, including two etiologic and three phenotypic criteria. The etiologic criteria were (1) reduced food intake or assimilation and (2) disease burden/inflammatory condition. The phenotypic criteria included not only low body mass index (BMI) and non-volitional weight loss but also reduced muscle mass reflecting changes in body composition [4].

According to the GLIM algorithm, the first step in diagnosing malnutrition is risk screening with a validated tool. The Mini Nutritional Assessment—Short Form (MNA-SF) is among the most commonly used screening tools in older adults [5,6,7]. Other questionnaires, such as the Malnutrition Universal Screening Tool (MUST) and Nutritional Risk Score (NRS-2002), can be used interchangeably [8,9,10]. The accuracy of the GLIM diagnostic algorithm [11,12,13] using various screening questionnaires has been intensively studied [14,15,16,17]. Some authors questioned the use of a screening tool as a first step in the algorithm. They pointed out that obtaining a score indicating normal nutritional status disqualifies a patient from consequent assessment of etiologic and phenotypic criteria. In reality, such a person may fulfil these criteria. They suggested using clinical suspicion of malnutrition interchangeably with the positive result of a screening test [18,19]. As patients with COPD have a particularly high risk of malnutrition [20,21,22], it may be assumed that all of them have a clinical suspicion of malnutrition. Consequently, all of them should be offered a full diagnostic of malnutrition.

Our study aimed to assess the diagnostic performance of three screening tools (MNA-SF, MUST, and NRS-2002) used in the GLIM algorithm in older patients with COPD. Additionally, we evaluated the agreement between these tools in the diagnostics of malnutrition. Our study is the first such analysis in subjects with COPD to the best of our knowledge.

## 2. Materials and Methods

**Figure 1:**
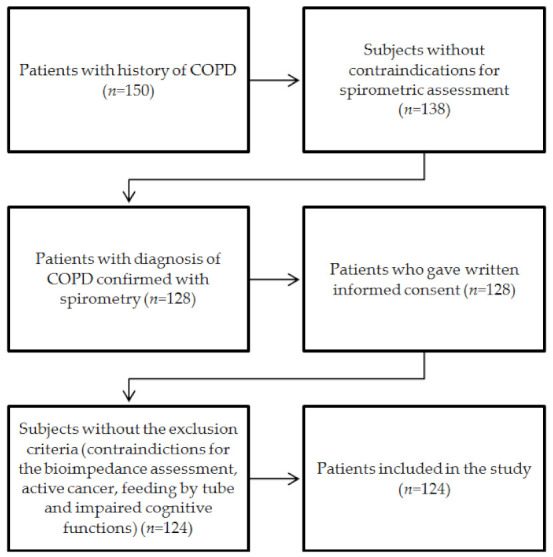
The process of selection and recruitment of patients. Notes: COPD, chronic obstructive pulmonary disease.

We performed a cross-sectional study in patients aged at least 60 years (age range: 60–86 years) with a COPD diagnosis based on the Global Initiative for Chronic Obstructive Lung Disease (GOLD) [23] criteria, who were hospitalized in Pulmonary Rehabilitation Ward (Great Poland Centre of Pulmonology and Thoracic Surgery) between September 2019 and November 2020. The inclusion criteria were preserved cognitive abilities and written informed consent to participate in the study. Subjects with contraindications for body composition analysis with the bioimpedance method (inability to maintain a standing position, cardiac pacemaker, metal objects, oedemas), patients feeding by tube, and patients with active cancer were not enrolled. The process of selection and recruitment of patients to our study is shown as a flowchart (Figure 1). The 150 subjects with history of COPD were admitted to the Pulmonary Rehabilitation Ward during the study period. Spirometry was not performed in 12 (8.0%) of them (owing to recent myocardial infarction, recent vascular surgery, recent ophthalmology surgery or history of retinal detachment). Of 128 patients with diagnosis of COPD confirmed with spirometry, 4 (3.1%) subjects met at least one study exclusion criterion. Finally, 124 individuals were included in our analysis.

The study was conducted under the Declaration of Helsinki. Its protocol was approved by the Bioethical Committee of the Poznan University Medical Sciences, Poland (approval No:888/19).

We took the results of the respiratory functional tests for our analysis from the hospital database. We assessed participants’ nutritional status with the three examined screening questionnaires (MNA-SF, MUST, and NRS-2002). Regardless of their results, we performed full malnutrition diagnostics following the GLIM algorithm in all subjects. Additionally, we performed diagnostics for sarcopenia according to the European Working Group on Sarcopenia in Older People (EWGSOP2) recommendations [24]. Data on comorbidities were taken from patients’ medical records. The sociological data were taken during a personal interview with participants.

### 2.1. Respiratory Functional Tests

All subjects underwent spirometry (LUNGTEST 1000, MES). Based on the GOLD recommendations, subjects with FEV1/FVC (forced expiratory volume in 1 second/forced vital capacity) below 70 were diagnosed with COPD [23]. The severity of the COPD was classified as follows:

- GOLD 1 (mild obturation) FEV1 ≥ 80%
- GOLD 2 (moderate obturation) FEV1 ≥ 50%
- GOLD 3 (severe obturation) FEV1 ≥ 30%
- GOLD 4 (very severe obturation) FEV1 < 30%

As there were few subjects in group GOLD 1 and GOLD 4, pooled categories GOLD1+GOLD2 (GOLD1+2) and GOLD3+GOLD4 (GOLD3+4) were used in further analysis.

### 2.2. Assessment of Cognitive Performance

Patients’ cognitive performance was assessed with the Abbreviated Mental Test Score (AMTS). It consists of ten items scoring one point each. Subjects who scored <7 (indicating significant cognitive dysfunction) were excluded from our analysis [25].

### 2.3. Diagnostics of Malnutrition

The assessment of nutritional status (with all questionnaires) was performed by one person (a certified dietician).The anthropometric assessment (total body mass, content of water, amount and percentage of fat tissue and lean body mass) was performed with the dual frequency InBody 120 analyzer (Biospace, Seoul, South Korea). The device uses 8 adhesive electrodes to measure segmental impedance (right arm, left arm, trunk, right leg, left leg). Subjects were asked to take off clothing and jewellery, stand barefoot on electrodes and grasp the hand electrodes. The measurements were taken after at least 2 h fasting.

#### 2.3.1. Mini Nutritional Assessment—Short Form Questionnaire

The Mini Nutritional Assessment—Short Form questionnaire contains six items: decrease in food intake, weight loss during the preceding three months, mobility, psychological stress or acute disease during the preceding three months, neuropsychological problems, and BMI. The maximum score is 14, and a score of fewer than 12 points indicates a risk of malnutrition [5].

#### 2.3.2. Malnutrition Universal Screening Tool (MUST)

The MUST questionnaire contains BMI, percentage unplanned weight loss in the preceding 3–6 months, and no nutritional intake for more than five days owing to an acute illness. The maximum score is 6, and a score of 1 or more indicates a risk of malnutrition [26].

#### 2.3.3. Nutritional Risk Screening 2002 (NRS-2002)

The NRS-2002 questionnaire refers to two aspects of malnutrition: deterioration of nutritional status (weight loss, low BMI, reduced dietary intake) and increased requirements due to acute and chronic illnesses. Subjects aged 70 and more are given an additional 1 point. The maximum score is 7, and a score ≥3 indicates a risk of malnutrition and the necessity for nutritional intervention [26].

#### 2.3.4. Global Leadership Initiative on Malnutrition (GLIM) Criteria

##### Phenotypic Criteria

The following phenotypic GLIM criteria were used:

1. Low BMI: <20 kg/m^2^ in persons aged below 70 years and <22 kg/m^2^ in subjects aged 70 or more years

2. Low muscle mass was diagnosed based on the bioimpedance analysis of the body composition. We used appendicular lean mass (ALM) to calculate the ALM index (a ratio of ALM (in kg) to squared body height (in m^2^)). Low muscle mass (LMM) was diagnosed in subjects with an ALM index below the previously estimated Polish cut-off points (5.6 kg/m^2^ in women and 7.4 kg/m^2^ in men) [27].

3. Unintended weight loss >5% body mass in the past six months, or >10% in a period longer than six months.

##### Etiologic Criteria

We assumed that COPD fulfils the etiologic criterion of chronic disease with severe systemic inflammation. Consequently, we diagnosed malnutrition in all participants with at least one phenotypic criterion.

### 2.4. Diagnostics of Sarcopenia

We measured upper limb muscle strength with a hand dynamometer (Saehan, Changwon, South Korea) in all participants. We assumed the values <16 kg in women and <27 kg in men indicate low upper limb muscle strength. Additionally, we assessed lower limb strength with the chair stand test (CST). Low lower limb muscle strength was diagnosed in subjects with the CST time >15 s. Participants with low upper and/or lower limb strength were classified as at risk of sarcopenia [24].

The diagnosis of sarcopenia in patients at risk was confirmed based on the ALM index.

### 2.5. Statistical Analysis

Continuous data were described with mean and standard deviation, and categorical variables with numbers (*n*) and percentage (%).

The differences between the four subgroups were assessed with the following tests:

- Analysis of variance (ANOVA)—for samples with normal distribution and homogeneity of variance;
- Kruskal–Wallis test—for samples not fulfilling the homogeneity of variance criterion.

The differences between the two subgroups were assessed with:

- Student *t*-test—for samples with normal distribution and homogeneity of variance;
- Mann–Whitney U test—for samples not following a normal distribution.

Categorical data were evaluated with Pearson’s chi-squared test.

In order to assess diagnostic performance of the MNA-SF, MUST, and NRS-2002 questionnaires against the GLIM criteria, we calculated sensitivity and specificity (>80% good, 50–80% moderate, <50% poor), positive predictive value (PPV), negative predictive value (NPV), area under the ROC curve (AUC; >0.8 good performance, 0.6–0.8 fair performance, <0.6 poor performance) and kappa coefficient (>0.8 very good level of agreement, 0.61–0.8 good level of agreement, 0.41–0.6 moderate level of agreement, 0.21–0.4 fair level of agreement, and <0.2 poor level of agreement) [28].

*p*-value < 0.05 was considered statistically significant. Statistical analyses were performed with STATISTICA 10 PL (StatSoft, Cracow, Poland).

## 3. Results

### 3.1. Study Population Characteristics

We included 124 subjects aged 60 years or more (mean age: 69.4 ± 6.1 years) admitted to the Pulmonary Rehabilitation Ward (Great Poland Centre of Pulmonology and Thoracic Surgery). The length of stay on the ward was 21 days in all patients. Men constituted 59.7% of the study sample. All participants were of Polish nationality. They were taking from 1 to 20 prescribed medications (mean 8.3). Seventy-nine percent of subjects had vocational or secondary education. Nearly two-thirds of the patients (64.3%) were living in urban areas, and 78.6% of them had households consisting of at least two persons. In addition to COPD, most participants had other medical conditions. The most frequent comorbidities were hypertension (62.9%), diabetes (30.6%), cardiovascular disease (29.0%) and dyslipidemia (22.6%).

Table 1 shows the characteristics of study participants according to the severity of COPD (GOLD 1+2 and GOLD 3+4). The results of the MNA-SF and NRS-2002 questionnaires did not differ between the two subgroups, while in the group GOLD 3+4, more subjects gained a MUST score indicative of impaired nutritional status. More patients with severe or very severe obstruction had low ALM index (*p* = 0.0047) than individuals with mild to moderate obstruction. They also had lower weight (*p* = 0.0048), BMI (*p* = 0.0046), skeletal muscle mass (*p* = 0.0071), and free fat mass (*p* = 0.0117) as compared to the GOLD1+2 group.

**Figure 2:**
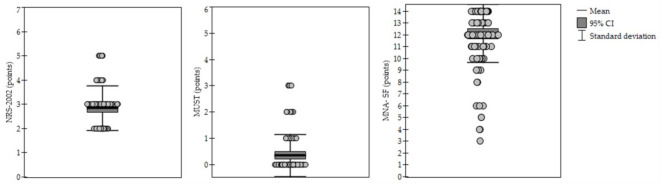
The distribution of scores for the screening questionnaires. Notes: NRS-2002, Nutritional Risk Score; MUST, Malnutrition Universal Screening Tool; MNA-SF, Mini Nutritional Assessment—Short Form.

Figure 2 shows the distribution of scores for the screening questionnaires.

### 3.2. Malnutrition According to Various Diagnostic Methods

Table 2 shows the results of spirometric assessments and anthropometric characteristics of patients diagnosed with malnutrition based on our study’s four diagnostic methods. Subjects with malnutrition diagnosed based on the MUST questionnaire and the GLIM criteria had the lowest body mass (*p* = 0.0318) and BMI (*p* = 0.0249). Other anthropometric parameters and functional test results did not differ between the individuals with malnutrition diagnosed with various questionnaires. The prevalence of sarcopenia was similar in all four groups, and all subjects with sarcopenia fulfilled the GLIM criteria for malnutrition.

**Figure 3:**
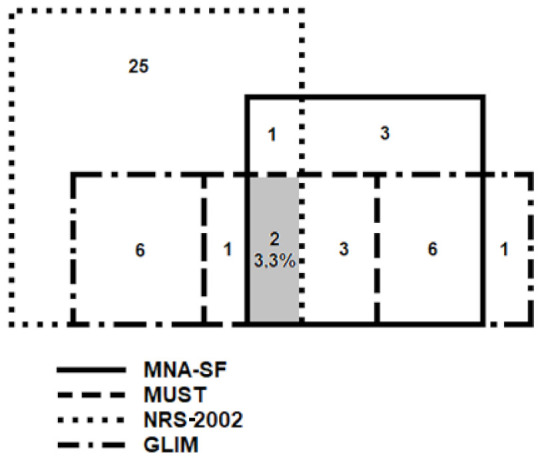
Number of patients with malnutrition diagnosed based on results of the MNA-SF, MUST, NRS-2002 questionnaires and the GLIM criteria. Notes: MNA-SF, Mini Nutritional Assessment— Short Form; MUST, Malnutrition Universal Screening Tool; NRS-2002, Nutritional Risk Score; GLIM, Global Leadership Initiative on Malnutrition. The grey area denotes overlapping of the MNA-SF, MUST, NRS-2002, and GLIM results for malnutrition.

Figure 3 shows an overlap between the results of the screening questionnaires and the GLIM diagnostic criteria. The proportion of malnourished participants varied from 18.5% for the MUST questionnaire to 27.4% for the MNA-SF and 57.3% for the NRS-2002 score. Based on the GLIM criteria, malnutrition was diagnosed in 48 subjects (38.7%). The results of the four assessed tools were in agreement in 24 (19.4%) participants without malnutrition and only eight subjects (6.5%) with malnutrition.

### 3.3. Diagnostic Performance of the MNA-SF, MUST, and NRS-2002 Questionnaires

Table 3 shows the assessed questionnaires’ sensitivity, specificity, positive and negative predictive value, accuracy, AUC, and kappa coefficient. Only the MNA-SF tool had fair sensitivity (58.3%), while the MUST and NRS-2002 questionnaires had poor sensitivity (47.9% for both questionnaires). The sensitivity of the MUST tool was higher in subjects with severe and very severe obstruction (fair; 58.6%). The MUST questionnaire had the highest specificity (100%), as compared to the MNA-SF (good; 92.1%) and NRS-2002 (poor; 36.8%). The accuracy was good for the MNA-SF (AUC 0.84), fair for the MUST (AUC 0.74), and poor for the NRS-2002 (AUC 0.41).

**Figure 4:**
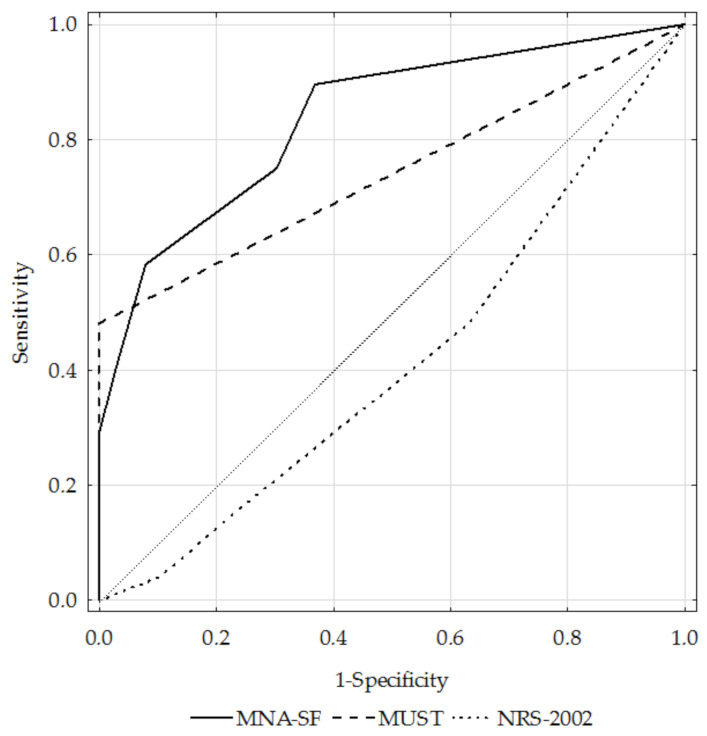
ROC curves for the MNA-SF, MUST, and NRS-2002 questionnaires against the GLIM criteria in the total study population.Notes: MNA-SF, Mini Nutritional Assessment—Short Form; MUST, Malnutrition Universal Screening Tool; NRS-2002, Nutritional Risk Score.

**Figure 5:**
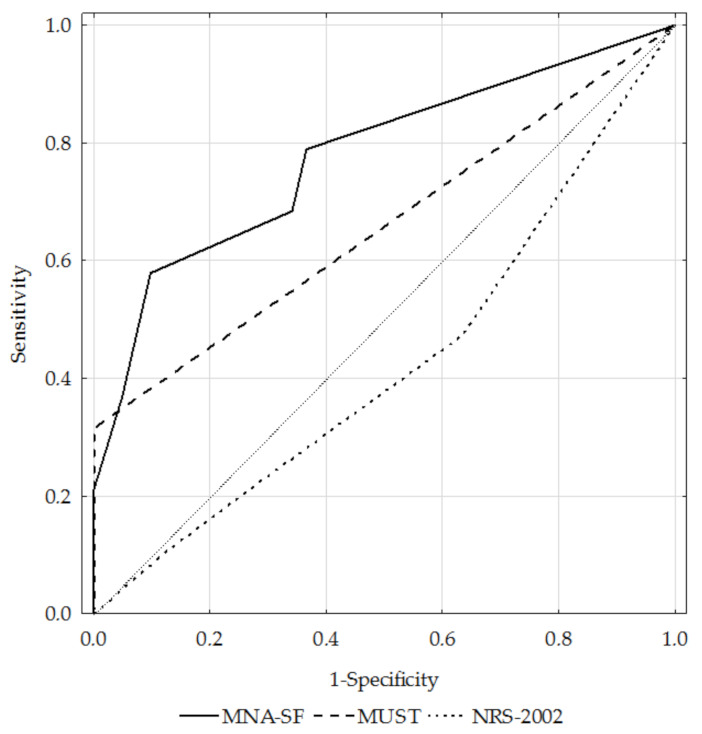
ROC curves for the MNA-SF, MUST, and NRS-2002 questionnaires against the GLIM criteria in the GOLD1+2 subgroup. Notes: MNA-SF, Mini Nutritional Assessment—Short Form; MUST, Malnutrition Universal Screening Tool; NRS-2002, Nutritional Risk Score; GLIM, Global Leadership Initiative on Malnutrition; GOLD, Global Initiative for Chronic Obstructive Lung Disease.

**Figure 6:**
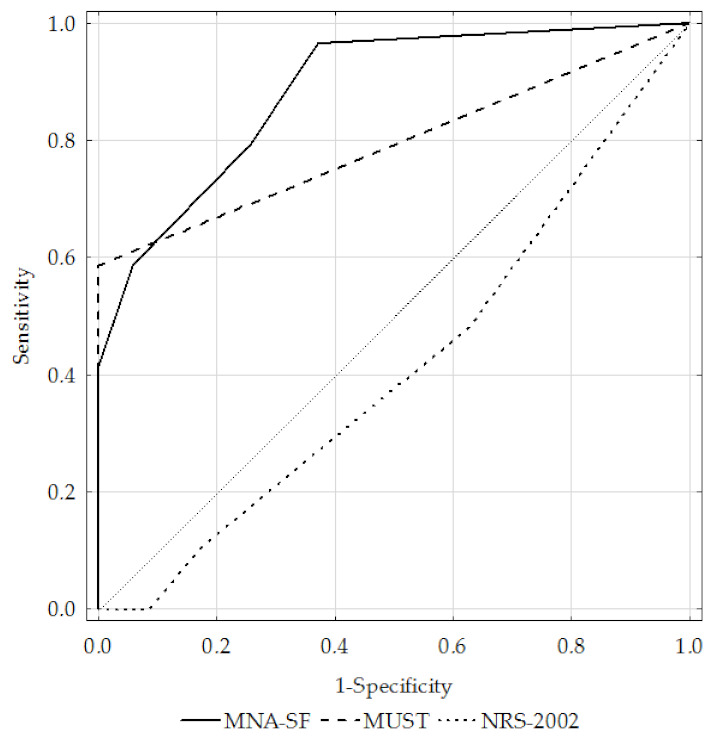
ROC curves for the MNA-SF, MUST, and NRS-2002 questionnaires against the GLIM criteria in the GOLD3+4 subgroup. Notes: MNA-SF, Mini Nutritional Assessment—Short Form; MUST, Malnutrition Universal Screening Tool; NRS-2002, Nutritional Risk Score; GLIM, Global Leadership Initiative on Malnutrition; GOLD, Global Initiative for Chronic Obstructive Lung Disease.

Figure 4 presents the ROC curves for the MNA-SF, MUST, and NRS-2002 questionnaires against the GLIM criteria in the total study sample, while Figure 5 and Figure 6 present them for the GOLD1+2 and GOLD3+4 subgroups, respectively.

## 4. Discussion

Volkert et al. [2] have recently specified seven common diseases that concomitantly increase the energy demand, decrease food intake and bioavailability, and thus increase the risk of malnutrition in elderly subjects. Chronic obstructive pulmonary disease was listed among these conditions [2]. To the best of our knowledge, our study was the first attempt to determine the best screening tool for malnutrition in elderly subjects with COPD. Malnutrition is a recognized negative prognostic factor in such patients, and interventions to improve nutritional status effectively improve general health status [3,29,30,31].

Despite the general acceptance of the GLIM diagnostic criteria for malnutrition published in 2018, the use of screening tools in the first step of diagnostics is the subject of much controversy [18,19,32,33]. Our results show that the use of the MNA-SF questionnaire as a screening tool would preclude the diagnosis of malnutrition in 20 subjects (41.7%), while the application of the MUST or NRS-2002 tool in 25 (52.1%) of patients (different individuals for both questionnaires).

Dávalos-Yerovi et al. [34] have suggested that the use of the etiologic criterion “any disease burden or inflammatory condition” in the GLIM algorithm may result in a higher percentage of subjects with a diagnosis of malnutrition. Chronic obstructive pulmonary disease may fulfil such an etiologic condition. However, the GLIM recommendations do not specify if it should be applied to all patients with COPD or only to individuals with the disease exacerbation. We assumed in our analysis that all patients with COPD fulfil this etiologic criterion. Moreover, weight or muscle mass loss is often observed in COPD subjects, fulfilling the phenotypic criteria [34]. Concomitant fulfilling of a phenotypic and etiologic criterion indicates malnutrition, even if the results of a screening tool are negative.

The screening questionnaires assessed in our study have been previously examined in other populations. Sanchez-Rodriguez et al. [32] observed that more than half (60.4%) of community-dwelling older adults (mean age: 73.2 ± 6.05 years) with malnutrition according to the GLIM criteria had MNA-SF score of at least 12 [32]. Similarly, in our previous work [19] involving 273 community-dwelling elderly subjects (mean age: 72.1 ± 7.7 years), etiologic and phenotypic criteria for malnutrition were fulfilled in 103 individuals, of whom 41% (*n* = 42) had a negative screening result with the MNA-SF [19].

These findings [19,32] question the value of MNA-SF as a screening tool for malnutrition in elderly subjects when the GLIM algorithm is used, in opposition to earlier reports [5,6]. Other screening tools have also been put under criticism. Bellanti et al. [17] assessed the diagnostic performance of the MUST, NRS-2002, and Subjective Global Assessment (SGA) in older inpatients (*n* = 152, mean age 78.3 ± 7.6 years). Similarly to our results, the MUST questionnaire had a higher diagnostic performance with an accuracy of 73.7% (MUST accuracy in our study was 79.8%) as compared to NRS-2002 with the accuracy of 62.5% (41.1% in our study); of note, none of the tools had good diagnostic performance [17]. It should be emphasized that unlike MNA-SF and MUST, which were elaborated to screen subjects living in various places (community-dwelling, institutionalized), the NRS-2002 questionnaire was worked up to assess the effectiveness of nutritional interventions in hospitalized adults. Therefore, the NRS-2002 is probably not well suited for pulmonary rehabilitation patients (who constituted our study population).

Overlooking malnutrition, particularly in elderly individuals, may lead to sarcopenia. The concomitance of these conditions is defined as malnutrition-sarcopenia syndrome (MSS). Hu et al. [35], in a study involving 453 elderly hospitalized subjects (mean age 79.0 ± 7.8 years), observed that patients with MSS has double the risk of death as compared to individuals with malnutrition or sarcopenia alone (HR for MSS: 4.78; HR for malnutrition: 2.62; HR for sarcopenia: 1.66) [35]. In our study, all subjects with sarcopenia fulfilled the GLIM criteria for malnutrition, confirming the frequent concomitance of these conditions. However, many individuals with sarcopenia had a negative result of a screening test for malnutrition, which further emphasizes the necessity to perform the complete diagnostics for malnutrition in elderly subjects with COPD, regardless of the screening results.

There are some limitations to our study. As an objective assessment of weight changes within the past 12 months was impossible, we based on patients’ declarations. Similarly, information about the reduction in dietary intake was not derived on calculations of nutritional demand and assessment with a diary but was declarative only. This limitation did not influence the prevalence of malnutrition according to the GLIM criteria, as COPD in all subjects fulfilled the other etiologic criterion (any disease burden or inflammatory condition). As our study has a cross-sectional design, any causal conclusion cannot be drawn. The trial was conducted in one district, so its findings could not be generalized.

To the best of our knowledge, our study was the first attempt to assess the relationship between malnutrition diagnosed with various screening tools and the severity of COPD in elderly subjects. It fills a gap in the literature and emphasizes the importance of a full diagnostic of malnutrition in these patients.

## 5. Conclusions

Our study demonstrates unsatisfactory sensitivity against the GLIM criteria of the MNA-SF, MUST and NRS-2002 questionnaires. The use of various screening questionnaires in the first step of the GLIM algorithm in elderly subjects with COPD yielded conflicting results. These findings have important clinical implications. Considering the negative health consequences of malnutrition, a full diagnostic including GLIM etiologic and phenotypic criteria should be recommended in all elderly patients with COPD, regardless of the screening results.

## Figures and Tables

**Table 1:** Characteristics of study population according to the severity of COPD.

		GOLD 1+2 *n* = 60	GOLD 3+4 *n* = 64	*p*-Value
Age (years)		69.8 ± 6.5	69.0 ± 5.7	0.4832
MNA-SF	Risk of malnutrition	15 (25.0)	19 (29.7)	0.5587
	No risk of malnutrition	45 (75.0)	45 (70.3)	
MUST	Risk of malnutrition	6 (10.0)	17 (26.6)	0.0177
	No risk of malnutrition	54 (90.0)	47 (73.4)	
NRS-2002	Risk of malnutrition	35 (58.3)	36 (56.3)	0.8147
	No risk of malnutrition	25 (41.7)	28 (43.8)	
Height (cm)		166.6 ± 9.4	166.3 ± 9.2	0.8641
Weight (kg)		82.8 ± 19.1	73.5 ± 21.1	0.0048
BMI (kg/m^2^)		29.8 ± 6.2	26.4 ± 6.6	0.0046
BFM (kg)		28.3 ± 12.4	24.2 ± 13.0	0.0591
SMM (kg)		30.2 ± 6.7	26.9 ± 6.5	0.0071
PBF (%)		33.1 ± 9.6	31.2 ± 10.2	0.2901
FFM (kg)		47.5 ± 11.0	42.4 ± 10.9	0.0117
Low ALM index		7 (11.7)	22 (34.4)	0.0028
FEV1/FVC EX		59.4 ± 7.6	43.2 ± 11.4	<0.0001
FEV1		66.7 ± 13.2	35.6 ± 7.1	<0.0001

Notes: Values are presented as numbers (%) or mean ± standard deviation for descriptive analyses. GOLD: Global Initiative for Chronic Obstructive Lung Disease; MNA-SF, Mini Nutritional Assessment—Short Form; MUST, Malnutrition Universal Screening Tool; NRS-2002, Nutritional Risk Score; BMI, body mass index; BFM, body fat mass; SMM, skeletal muscle mass; PBF, percent body fat; FFM, free fat mass; ALM index, appendicular lean mass index; FEV1/FVC EX, forced expiratory volume in 1 s/forced vital capacity; FEV1, forced expiratory volume in 1 s.

**Table 2:** Anthropometric characteristics and clinical data of subjects with malnutrition diagnosed based on four different diagnostic methods.

	GLIM, *n* = 48	MNA-SF + GLIM, *n* = 28	MUST + GLIM, *n* = 23	NRS-2002 + GLIM, *n* = 23	*p*-Value
Age	69.1 ± 6.3	69.4 ± 6.0	68.8 ± 5.1	69.8 ± 6.7	0.9509
AMTS	9.5 ± 0.7	9.6 ± 0.6	9.7 ± 0.7	9.7 ± 0.6	0.7974
Sex					0.3503
Women	20 (41.7)	16 (57.1)	12 (52.2)	8 (34.8)	
Men	28 (58.3)	12 (42.9)	11 (47.8	15 (65.2)	
GOLD					
1+2	19 (39.6)	11 (39.3)	6 (26.1)	9 (39.1)	0.7006
3+4	29 (60.4)	17 (60.7)	17 (73.9)	14 (60.9)	
FEV1/FVC EX	48.1 ± 10.9	49.3 ± 10.9	47.1 ± 11.1	46.3 ± 10.9	0.7799
FEV1	48.9 ± 20.2	49.3 ± 19.5	43.8 ± 18.6	48.5 ± 21.3	0.7811
6MWT (m)	317.1 ± 134.2	281.0 ± 143.4	286.8 ± 152.7	332.8 ± 145.4	0.5403
Height (cm)	165.4 ± 9.4	162.8 ± 10.2	163.7 ± 8.6	165.1 ± 10.5	0.5409
Weight (kg)	66.2 ± 18.6	61.0 ± 19.2	55.0 ± 12.9	67.5 ± 18.5	0.0318
BMI (kg/m^2^)	24.0 ± 5.8	22.8 ± 6.0	20.4 ± 3.6	24.6 ± 5.6	0.0249
BFM (kg)	19.3 ± 11.1	17.6 ± 11.1	13.5 ± 6.8	20.4 ± 10.9	0.0758
SMM (kg)	25.5 ± 6.3	23.4 ± 6.3	22.3 ± 4.6	25.6 ± 6.2	0.0745
PBF (%)	27.6 ± 9.8	26.9 ± 10.0	23.6 ± 7.4	29.0 ± 9.0	0.2336
FFM (kg)	40.0 ± 10.8	36.3 ± 10.9	34.4 ± 8.0	40.5 ± 10.8	0.0706
Low ALM index	29 (60.4)	19 (67.9)	18 (78.3)	15 (65.2)	0.5200
Sarcopenia	16 (33.3)	12 (42.9)	12 (52.2)	9 (39.1)	0.4923
No sarcopenia	32 (66.7)	16 (57.1)	11 (47.8)	14 (60.9)	

Notes: Values are presented as numbers (%) or mean ± standard deviation for descriptive analyses. AMTS, Abbreviated Mental Test Score; GOLD, Global Initiative for Chronic Obstructive Lung Disease; MNA-SF, Mini Nutritional Assessment—Short Form; MUST, Malnutrition Universal Screening Tool; NRS-2002, Nutritional Risk Score; FEV1/FVC EX, forced expiratory volume in 1 s/forced vital capacity; FEV1, forced expiratory volume in 1 s; 6MWT, 6 min walk test; BMI, body mass index; BFM, body fat mass; SMM, skeletal muscle mass; PBF, percent body fat; FFM, free fat mass; ALM index, appendicular lean mass index.

**Table 3:** Diagnostic performance of the MNA-SF, MUST, and NRS-2002 questionnaires.

		MNA-SF	MUST	NRS-2002
Sensitivity (%)	Total	58.3	47.9	47.9
	GOLD 1+2	57.9	31.6	47.4
	GOLD 3+4	58.6	58.6	48.3
Specificity (%)	Total	92.1	100.0	36.8
	GOLD 1+2	90.2	100.0	36.6
	GOLD 3+4	94.3	100.0	37.1
Positive predictive value (%)	Total	82.4	100.0	32.4
	GOLD 1+2	73.3	100.0	25.7
	GOLD 3+4	89.5	100.0	38.9
Negative predictive value (%)	Total	77.8	75.3	52.8
	GOLD 1+2	82.2	75.9	60.0
	GOLD 3+4	73.3	74.5	46.4
Accuracy (%)	Total	79.0	79.8	41.1
	GOLD 1+2	80.0	78.3	40.0
	GOLD 3+4	78.1	81.3	42.2
AUC	Total	0.84	0.74	0.41
	GOLD 1+2	0.78	0.66	0.42
	GOLD 3+4	0.89	0.79	0.41
Kappa coefficient	Total	0.533	0.530	0.140
	GOLD 1+2	0.510	0.387	0.131
	GOLD 3+4	0.545	0.608	0.143

Notes: MNA-SF, Mini Nutritional Assessment—Short Form; MUST, Malnutrition Universal Screening Tool; NRS-2002, Nutritional Risk Score; GOLD, Global Initiative for Chronic Obstructive Lung Disease; AUC, area under the curve.

## Data Availability

All relevant data are within the manuscript and are openly available in the Zenodo repository (DOI: 10.5281/zenodo.5833462).
